# *Albino seedling lethality 4*; Chloroplast 30S Ribosomal Protein S1 is Required for Chloroplast Ribosome Biogenesis and Early Chloroplast Development in Rice

**DOI:** 10.1186/s12284-021-00491-y

**Published:** 2021-05-27

**Authors:** Kunneng Zhou, Caijuan Zhang, Jiafa Xia, Peng Yun, Yuanlei Wang, Tingchen Ma, Zefu Li

**Affiliations:** Anhui Province Key Laboratory of Rice Genetics and Breeding, (Rice Research Institute Anhui Academy of Agricultural Sciences), Hefei, 230031 China

**Keywords:** Chloroplast rRNAs, *Oryza sativa*, Plastid-encoded genes, Plastid ribosomal proteins, Plastid transcription, 30S ribosomal protein S1

## Abstract

**Background:**

Ribosomes responsible for transcription and translation of plastid-encoded proteins in chloroplasts are essential for chloroplast development and plant growth. Although most ribosomal proteins in plastids have been identified, the molecular mechanisms regulating chloroplast biogenesis remain to be investigated.

**Results:**

Here, we identified albinic seedling mutant *albino seedling lethality 4* (*asl4*) caused by disruption of 30S ribosomal protein S1 that is targeted to the chloroplast. The mutant was defective in early chloroplast development and chlorophyll (Chl) biosynthesis. A 2855-bp deletion in the *ASL4* allele was verified as responsible for the mutant phenotype by complementation tests. Expression analysis revealed that the *ASL4* allele was highly expressed in leaf 4 sections and newly expanded leaves during early leaf development. Expression levels were increased by exposure to light following darkness. Some genes involved in chloroplast biogenesis were up-regulated and others down-regulated in *asl4* mutant tissues compared to wild type. Plastid-encoded plastid RNA polymerase (PEP)-dependent photosynthesis genes and nuclear-encoded phage-type RNA polymerase (NEP)-dependent housekeeping genes were separately down-regulated and up-regulated, suggesting that plastid transcription was impaired in the mutant. Transcriptome and western blot analyses showed that levels of most plastid-encoded genes and proteins were reduced in the mutant. The decreased contents of chloroplast rRNAs and ribosomal proteins indicated that chloroplast ribosome biogenesis was impaired in the *asl4* mutant.

**Conclusions:**

Rice *ASL4* encodes 30S ribosomal protein S1, which is targeted to the chloroplast. *ASL4* is essential for chloroplast ribosome biogenesis and early chloroplast development. These data will facilitate efforts to further elucidate the molecular mechanism of chloroplast biogenesis.

**Supplementary Information:**

The online version contains supplementary material available at 10.1186/s12284-021-00491-y.

## Background

Plastids have their own genome and transcriptional and translational systems. Plastid ribosomes are the main sites of plastid protein translation in higher plants. Nearly 120 proteins are translated in plastid ribosomes (Hiratsuka et al. 1989). Chloroplast biogenesis from a proplastid to mature chloroplast requires three steps and involves different regulatory genes (Kusumi et al. 2010). *FtsZ* is required for the first step: plastid DNA synthesis and plastid division (Vitha et al. 2001). *RpoTp*, *rpoA* and *rpoB*, are abundant in the second step: establishment of the plastid transcription/translation apparatus (De Santis-MacIossek et al. 1999; Kusumi et al. 2004). Genes *psaB*, *psbA*, *psbB*, *psbC*, *rbcL*, *rbcS*, *cab1R* and *cab2R* play important roles in the third step: activation of the photosynthetic apparatus (Hirai et al. 1985; Hwang and Tabita 1989; Matsuoka 1990; Nelson and Yocum 2006). In common with prokaryotes plastid ribosomes are composed of 50S large subunits and 30S small subunits. The large subunit combines 23S and 5S rRNA and contains 33 to 36 proteins. The small subunit consists of 16S rRNA and 21 to 25 proteins (Sharma et al. 2007; Yamaguchi et al., 2000, Yamaguchi and Subramanian, 2000).

Plastid ribosomes have important roles in plastid development and differentiation. Mutations in genes affecting plastid ribosomes can lead to disrupted embryonic development and albinism that is lethal following exhaustion of energy reserves in the endosperm of the parent seed. Maize PRPS17 was the first reported chloroplast ribosomal protein, mutation in which reduced the translation of plastid proteins and photosynthetic rate causing a light and temperature dependent lethal phenotype (Schultes et al. 2000). In addition, mutation of PRPS9, another member of the same gene family, caused death of the embryo and hence lack of germination (Ma and Dooner 2004; Qiu et al. 2018). Reverse genetics studies showed that chloroplast ribosomal small (PRPS9, 13 and 20) and large (PRPL1, 4, 6, 10, 13, 18, 21, 27, 28, 31 and 35) subunit proteins have essential roles in embryonic development and seed formation in Arabidopsis (Bryant et al. 2011; Hsu et al. 2010; Lloyd and Meinke 2012; Romani et al. 2012; Yin et al. 2012). Knockout of *PRPS2*, *S4*, *S18* and *L20* in tobacco affected protein synthesis and function of chloroplast ribosomes leading to cell death and leaf deformity (Rogalski et al. 2006, 2008). Rice *ASL1* and *ASL2* encode plastid ribosomal small subunit S20 and large subunit L21, respectively; *asl1* and *asl2* mutants suppressed chloroplast development and caused albinic seedlings (Gong et al. 2013; Lin et al. 2014). Loss of function of RPS20 in *E. coli*, the homologous protein of PRPS20, decreased ribosomal activity by modification of 16S rRNA, leading to inhibited assembly of 30s and 50s subunits in forming 70S ribosomes (Aulin et al. 1993). Highly down-regulated expression of 16S rRNA in an *asl1* mutant indicated that PRPS20 in rice has an important role in the accumulation of chloroplast ribosomes (Gong et al. 2013). A single amino acid variation in OsPRPL12 suppressed PEP transcription causing seedling albinism (Zhao et al. 2016).

However, not every ribosomal protein is essential for plant growth and development. Although mutations of some plastid ribosomal genes lead to decreased photosynthetic efficiency and plastid protein synthesis, they do not prevent whole-of-life processes of the plant. For example, mutations in AtPRPS17, L11 and L24 decrease the synthesis of plastid proteins and photosynthesis, but do not inhibit the basic activity of chloroplast ribosomes (Pesaresi et al. 2001; Romani et al. 2012). Knockout of *PRPL33* showed normal plastid translation and plant growth under natural conditions in tobacco but expressed leaf chlorosis and delayed growth following cold stress (Rogalski et al. 2008). *WLP1* was isolated to encode a PRPL13 protein in rice, and a *wlp1* mutant showed a white leaf and panicle phenotype at low temperature (Song et al. 2014). Previous reports suggested that L13 protein had important roles in the folding of 23S rRNA and assembly of 50S ribosomal large subunits (Maguire and Zimmermann 2001; Sharma et al. 2007). Loss of function of L13 in *E. coli* caused a lethal phenotype hence differing from the *wlp1* mutant, with an apparently weakened, rather than lethal variation of the *WLP1* gene (Song et al. 2014).

Ribosomal protein RPS1 was identified to recognize and bind multiple mRNAs to ribosomes at the initial stage of protein translation in Gram’s bacteria (Hajnsdorf and Boni 2012). A T-DNA mutant of *AtPRPS1* obtained by reverse genetics possessed only 8% of the wild-type transcript level, causing leaf chlorosis and delayed plant growth (Romani et al. 2012). Another study showed that PRPS1 interacted with GUN1 (Genomes uncoupled 1), and knockout of *GUN1* slowed down the degradation of PRPS1 protein in a *gun1prps1* mutant (Tadini et al. 2016).

In this study, we identified an *asl4* mutant that exhibited an albino seedling phenotype and died after the 3-leaf (L3) stage. The *ASL4* allele encodes 30S ribosomal protein S1 that is targeted to the chloroplast and affects the levels of plastid-encoded genes and proteins. PEP transcription and chloroplast ribosome biogenesis was suppressed in the *asl4* mutant. The data indicated that ASL4 protein is essential for establishment of the genetic system during early chloroplast development.

## Results

### Phenotypic Characteristics of the *asl4* Mutant

**Fig. 1:**
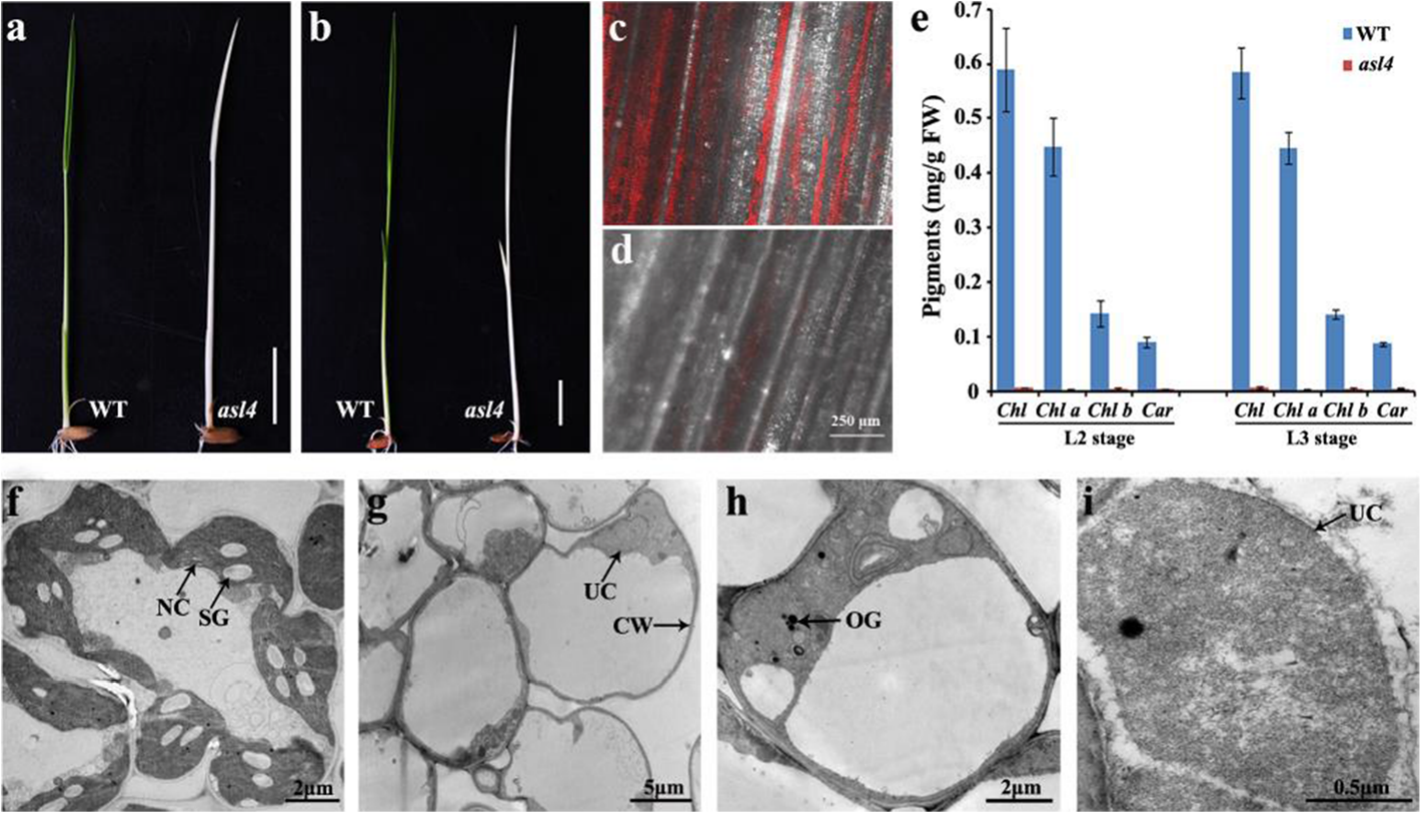
Phenotypic characteristics of the *asl4* mutant. **a** Phenotype of wild type and the *asl4* mutant at the L2 stage grown in the field. **b** Phenotype of wild type and the *asl4* mutant at the L3 stage. Confocal microscope observation of chlorophyll-containing cells in seedlings of wild type (**c**) and the *asl4* mutant (**d**). **e** Photosynthetic pigment determination in seedlings of wild type and *asl4* mutant at the L2 and L3 stages. Values are means ± SD from three independent replicates. TEM observations of chloroplasts in wild type (**f**) and *asl4* (**g**-**i**) seedlings at the L3 stage. *NC* normal chloroplast, *SG* starch granule, *UC* undifferentiated chloroplast, *CW* cell wall, *OG* osmiophilic plastoglobuli

The *asl4* albino mutant (Fig. 1a, b) was identified from a *N*-methyl-*N*-nitrosourea (MNU)-treated population of *Oryza sativa ssp. japonica* variety Nongyuan 238. Chl-containing cells were few in number in leaves of the *asl4* mutant compared to wild type (Fig. 1c, d). Consistent with the mutant phenotype, the *asl4* mutant could not synthesize Chl and carotenoids (Car) (Fig. 1e). To investigate the effect of the *asl4* mutation on chloroplast development, we examined the ultrastructure of chloroplasts by transmission electron microscopy (TEM). Wild-type chloroplasts contained well-developed lamellar structures with normally stacked grana and thylakoid membranes (Fig. 1f). In contrast, *asl4* mutant cells had few and small or undifferentiated chloroplasts with no thylakoid membranes (Fig. 1g-i). These data indicate that *ASL4* plays an essential role in early chloroplast development and plant growth.

### Cloning of the *ASL4* Gene

**Fig. 2:**
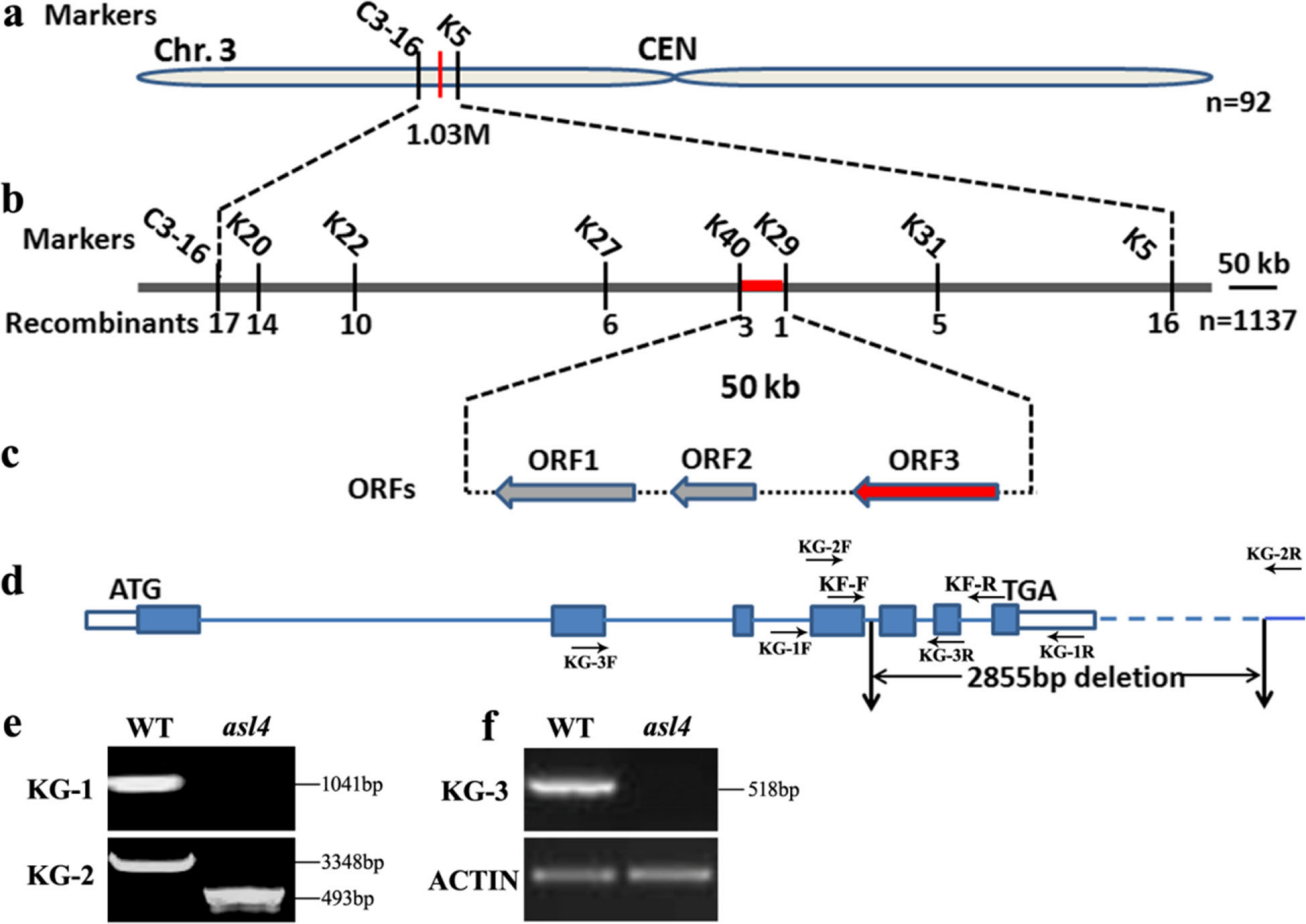
Map-based cloning of the *ASL4* locus. **a** The *ASL4* locus was initially mapped to a 1.03-Mb region between markers C3–16 and K5 on the short arm of chromosome 3. **b** *ASL4* was fine-mapped to a 50-kb region between markers K40 and K29 using 1137 F_2_ mutant seedlings. **c** Three ORFs were predicted in the region. **d** Struct. of *ASL4*. ATG and TGA indicate start and stop codons. *Blue boxes* indicate exons and the lines between them indicate introns. *White boxes* represent the 5′ and 3′ UTR. The location of the 2855-bp deletion in the *asl4* allele is indicated. PCR identification of genomic DNAs (**e**) and cDNA (**f**) between wild type and *asl4* mutant using primer pairs indicated in **d**. The *actin* gene was amplified as the control

The *asl4* mutant was preserved as a heterozygote, the progeny of which segregated 443 normal: 140 albino (χ^2^_3:1_ = 0.302, P_1df_ > 0.05), indicating that the *asl4* phenotype was conferred by a single recessive nuclear allele. A segregating F_2_ population from a cross of the *asl4* heterozygote (*ASL4*/*asl4*) and Nanjing11 was used for gene mapping. The *ASL4* locus was mapped to a 1.03-Mb region between insertion-deletion polymorphic (InDel) markers, C3–16 and K5, on the short arm of chromosome 3 (Fig. 2a). The *ASL4* locus was further delimited to a 50-kb region between markers K40 and K29 using 1137 albinic F_2_ individuals (Fig. 2b). Three open reading frames (ORFs) were predicted in the region from the RGAP database (http://rice.plantbiology.msu.edu/cgi-bin/gbrowse/rice/) (Fig. 2c). Sequence analysis demonstrated that the third ORF (*LOC_Os03g20100*) had a 2855-bp deletion from the 37th bp of intron 4 to the 2312th bp downstream of the TGA stop codon (Fig. 2d-f). The deletion caused a loss of 96 amino acid residues and added an extra of 29 amino acid residues resulting from the frame-shift translation (Fig. S1).

**Fig. 3:**
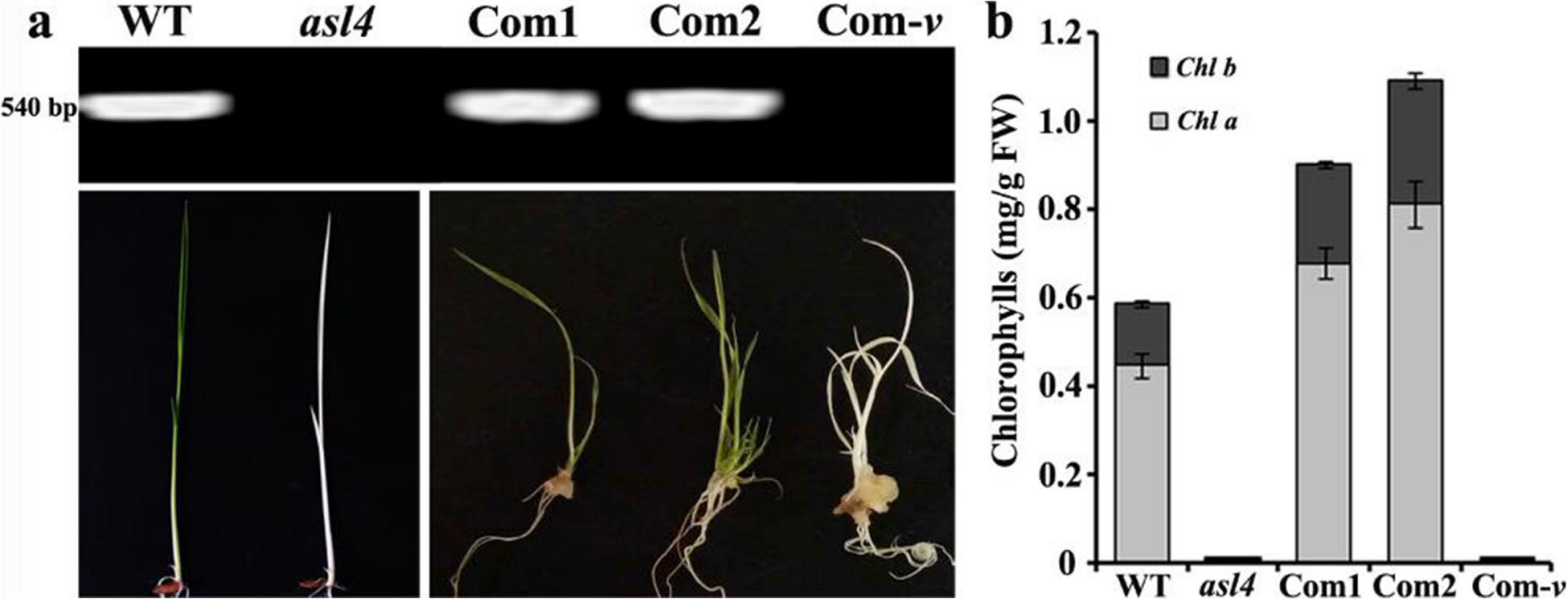
Complementation test of the *asl4* mutation. **a** Phenotypes of wild type, *asl4* mutant and transgenic plants. Primer KF indicated in Fig. 2d was used to distinguish positive and negative lines. 540 bp, size of the amplified product generated by primer KF. **b** Chlorophyll contents of wild type, *asl4* mutant and transgenic plants. Values are means ± SD from three independent repeats. Com1, Com2 and Com-v are two positive transgenic lines and transformation control with empty vector, respectively

To confirm whether mutation of *ASL4* was responsible for the *asl4* phenotype, expression vector p*GASL4* containing the entire wild-type *ASL4* genomic DNA was introduced into homozygous *asl4* calli cultured from the selfed progenies of *ASL4asl4* heterozygotes. Marker ‘KF’ was used to detect transgenic individuals (Fig. 2d; Fig. 3a). All positive plants complemented the *asl4* phenotype whereas the negative controls did not (Fig. 3a, b). These data provided evidence that *LOC_Os03g20100* corresponded to the *ASL4* locus.

### *ASL4* Encodes 30S Ribosomal Protein S1 that Is Targeted to the Chloroplast

The *ASL4* gene with 7 exons and 6 introns encoding a polypeptide of 402 amino acid residues was predicted to be plastid 30S ribosomal protein S1 (PRPS1) (Fig. 2d, Fig. S1). Sequence alignment showed only one copy of ASL4 containing a predicted RNA binding domain covering amino acid residues 254–323 and having extremely high similarity to PRPS1 proteins in other species (Fig. S2). The asl4 protein lacked an intact RNA binding domain that presumably disrupted the function of PRPS1 (Fig. S1). Phylogenetic analysis showed that PRPS1 orthologs exist in many photosynthetic organisms forming monocot and dicot subclades and likely having evolved from the bryophyta to angiosperms (Fig. S3).

**Fig. 4:**
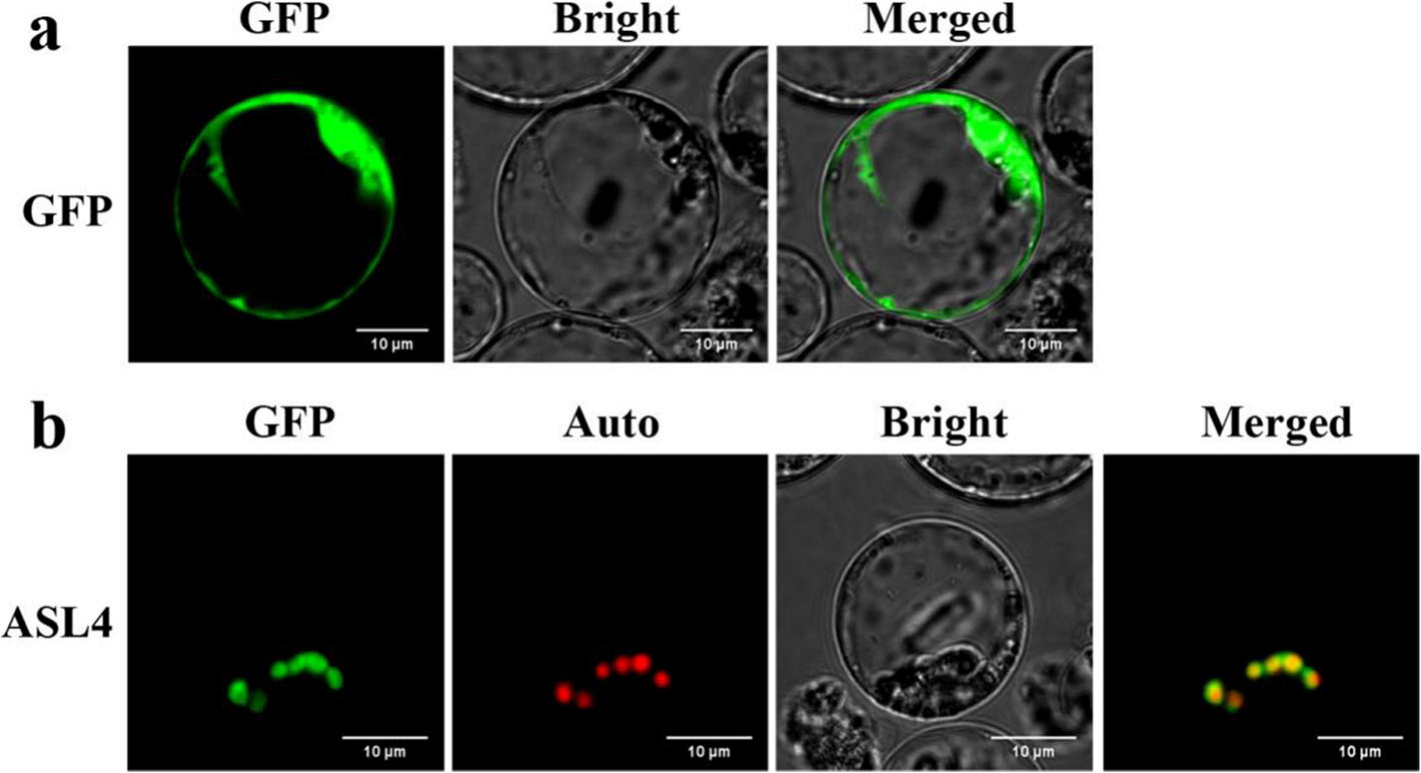
Subcellular location of ASL4 protein. **a** Free GFP signals in rice protoplasts. **b** ASL4-GFP signals were co-localized with chlorophyll autofluorescence in rice protoplasts. GFP, GFP signals of free GFP and ASL4; Auto, chlorophyll autofluorescence; Bright, bright field; Merged, merged images

ASL4 was predicted to be a plastid protein. To determine its localization, free green fluorescent protein (GFP) and a ASL4-GFP fusion plasmid were separately transformed into rice protoplasts. The free GFP was dispersed throughout the cytoplasm (Fig. 4a), whereas ASL4-GFP was merged with Chl autofluorescence (Fig. 4b), hence confirming that ASL4 was a chloroplast protein.

### Expression Analysis of *ASL4*

**Fig. 5:**
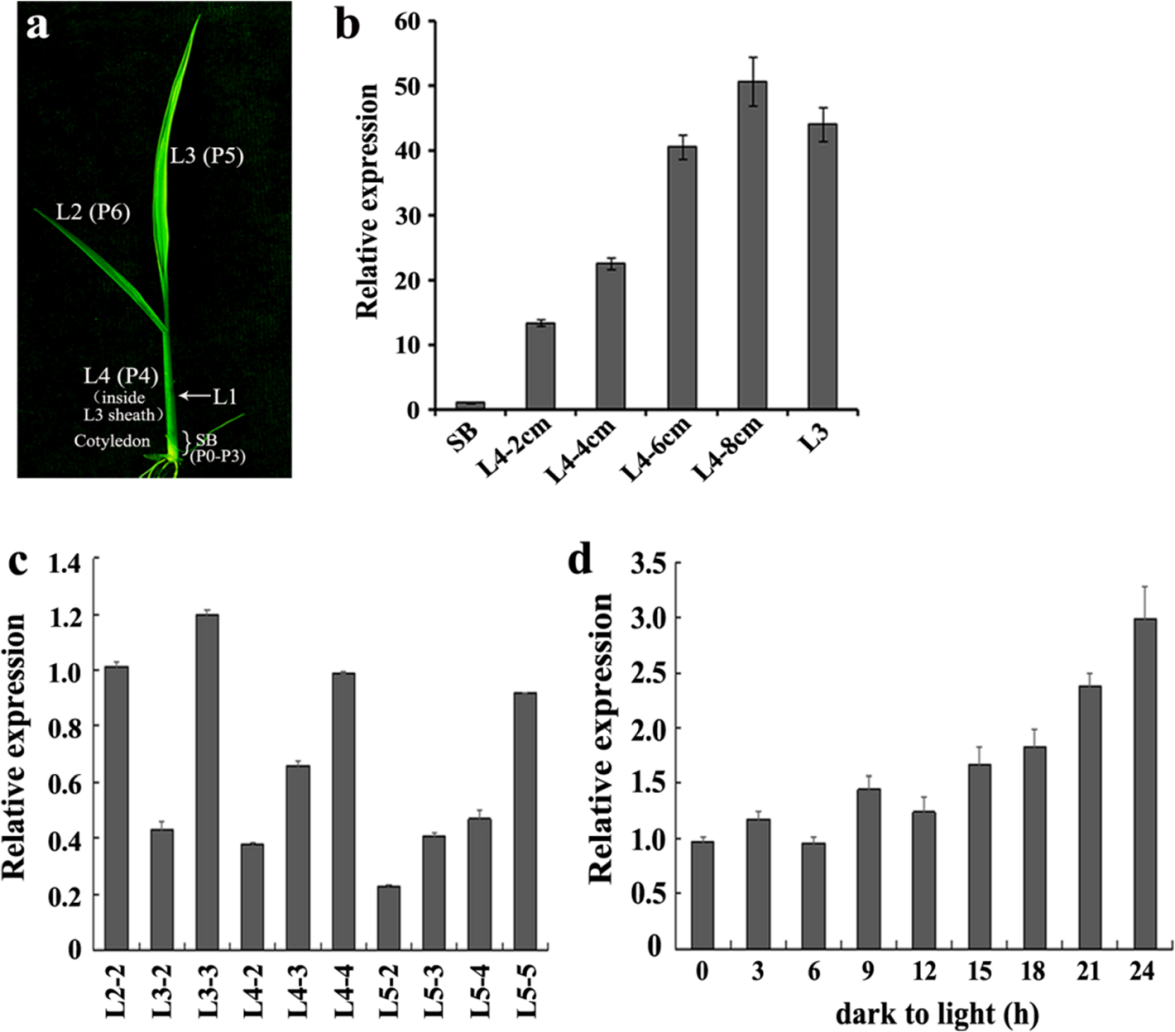
Expression analysis of *ASL4*. **a** Diagram of a L3 stage seedling when leaf 3 is fully expanded. SB indicates a 5 mm piece from the bottom of the shoot. L1-L4 represent leaves 1 to 4 in the L3 stage seedling. P0-P6 represent the developmental stages of leaf formation. **b** Expression of *ASL4* in different wild-type sections at the L3 stage seedling from the paddy field. L4–2 cm, 4 cm, 6 cm and 8 cm indicate the length of the L4 leaf. **c** Expression levels of *ASL4* in wild-type leaves at different stages. For example, L2–2 represent leaf 2 of a L2 stage seedling, L3–2 represent leaf 2 of a L3 stage seedling. **d** Expression analysis of *ASL4* during light-induced greening of wild-type seedlings. Wild-type seedlings were exposed to light for 3, 6, 9, 12, 15, 18, 21 and 24 h after 10 days of growth in darkness at 30 °C. The *ubiquitin* gene was used as internal control. Values are means ± SD from three independent replicates

Expression analysis showed that *ASL4* was constitutively expressed in various rice tissues, with extremely high levels in leaf blades and sheaths (Fig. S4). To detect growth stage-specific expression of *ASL4* during leaf development, we analyzed its expression levels in different leaf sections at stage L3. The *ASL4* gene was initially expressed in the shoot base (SB) and expression levels gradually increased as the L4 developed, and then decreased in L3 tissue, although there was still a high expression level (Fig. 5a, b). This indicated that *ASL4* participated in chloroplast biogenesis.

We also measured the *ASL4* transcript in leaf tissues at different seedling development stages. *ASL4* had highest expression levels in newly expanded leaves but levels declined with leaf aging (Fig. 5c). To identify the relationship between *ASL4* expression and light, we detected *ASL4* accumulation during light-induced greening of wild-type seedlings that had developed in darkness. *ASL4* mRNA levels increased with the extended time of illumination (Fig. 5d), indicating that light might play an important role in regulating *ASL4* expression.

### The *asl4* Mutant Is Defective in Plastid Transcription

**Fig. 6:**
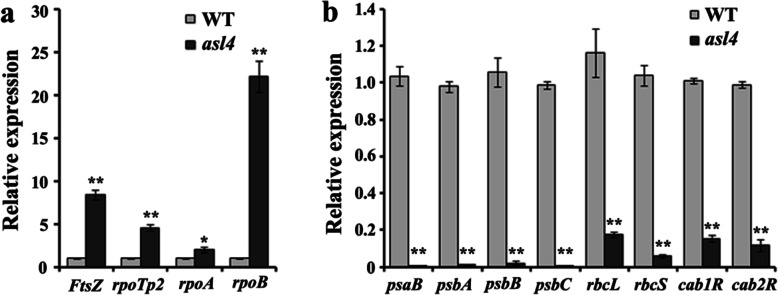
Expression levels of genes involved in chloroplast biogenesis. Expression levels of genes associated with the first and second (**a**) and third (**b**) steps of chloroplast biogenesis in wild-type and *asl4* mutant seedlings at the L3 stage. Data are means ± SD of three independent repeats. ** and *, indicate significance at *P* = 0.01 and *P* = 0.05, respectively, by Student’s *t* tests

Given the effect of the *ASL4* mutation on chloroplast development, we examined the expression levels of genes related to chloroplast biogenesis. Compared with the wild type, genes involved in the first (*FtsZ*) and second (*rpoTP2*, *rpoA* and *rpoB*) steps of chloroplast biogenesis were up-regulated in the *asl4* mutant (Fig. 6a), and genes required for the third step (*psaB*, *psbA*, *psbB*, *psbC*, *rbcL*, *rbcS*, *cab1R* and *cab2R*) were down-regulated (Fig. 6b). This suggested that mutation of *ASL4* impeded chloroplast development by disrupting the expression of genes involved in chloroplast biosynthesis. Down-regulated expression of PEP-dependent photosynthesis genes (such as *psaB*, *psbA* and *rbcL*) and up-regulated NEP-dependent housekeeping genes (*rpoA* and *rpoB*) (Fig. 6a, b) is a typical gene expression pattern resulting from impaired plastid transcription. Messenger-RNA levels of Chl biosynthesis-related genes (*PORA*, *HEMA1*, *YGL1*, *CHLI*, *CHLH* and *CHLD*) were obviously decreased in the *asl4* mutant compared to the wild type (Fig. S5).

### *ASL4* Affects Plastid-Encoded mRNA and Protein Levels

**Fig. 7:**
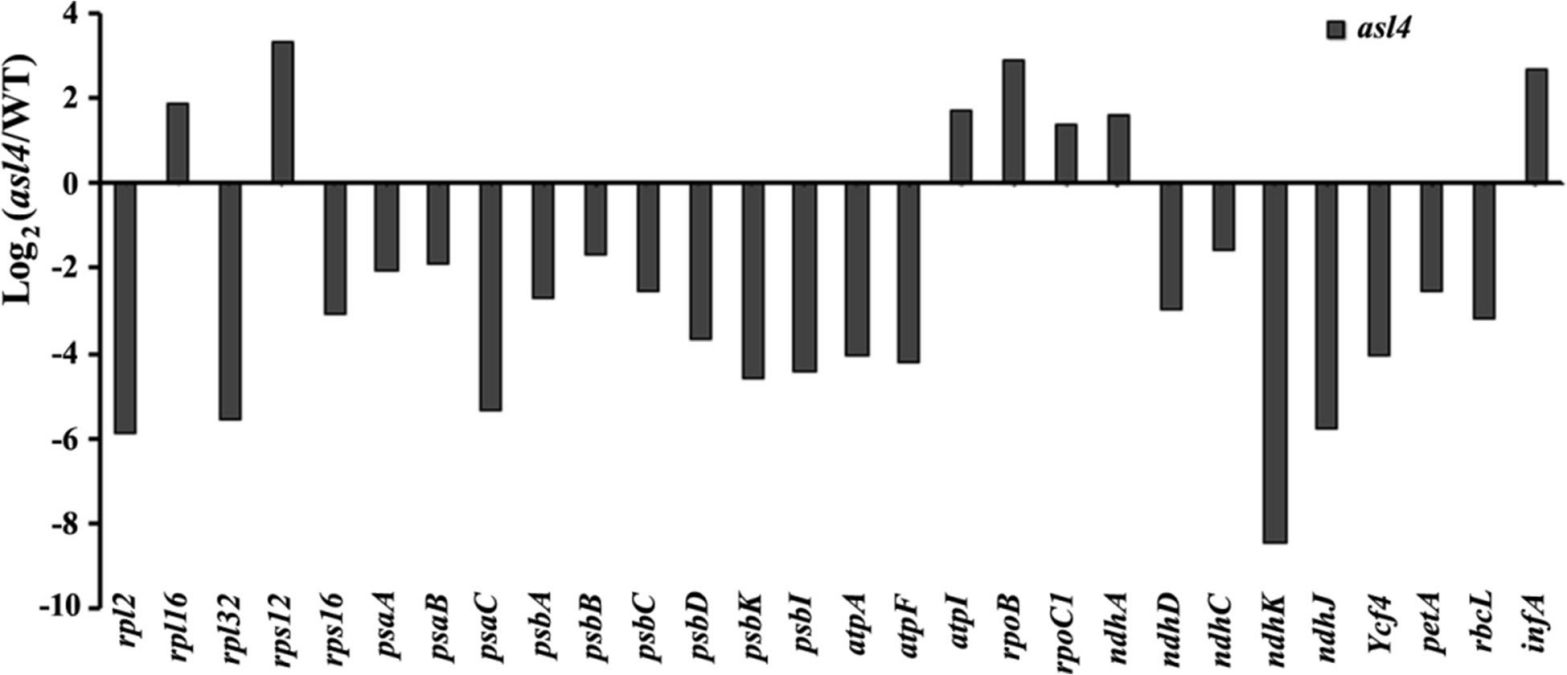
Transcripts of plastid-encoded genes detected by transcriptome analysis. Total RNA was isolated from wild-type and *asl4* mutant seedlings at the L3 stage and reverse-transcribed by random hexamer primers. The library was constructed and sequenced with an lllumina HiSeq 2000. Log_2_ (*asl4*/WT) indicates the log_2_ ratio of mRNA levels in *asl4* mutant compared to wild type

To further verify the effect of *ASL4* mutation on plastid transcription, we compared the expression levels of plastid-encoded genes in *asl4* mutant and wild type by transcriptome analysis. Expression of most of the tested plastid-encoded genes was lower in the mutant. The mRNA levels of Class I genes (e.g., *psaA*, *psaB*, *psbA*) transcribed by PEP, including photosystem I (PSI) and photosystem II (PSII), were lower in the *asl4* mutant, whereas Class III genes (e.g., *rpoB*, *rpoC1*) transcribed by NEP, and including RNA polymerase and ribosomal proteins, accumulated (Fig. 7). This was near-consistent with the results of qPCR (Fig. 6a, b).

**Fig. 8:**
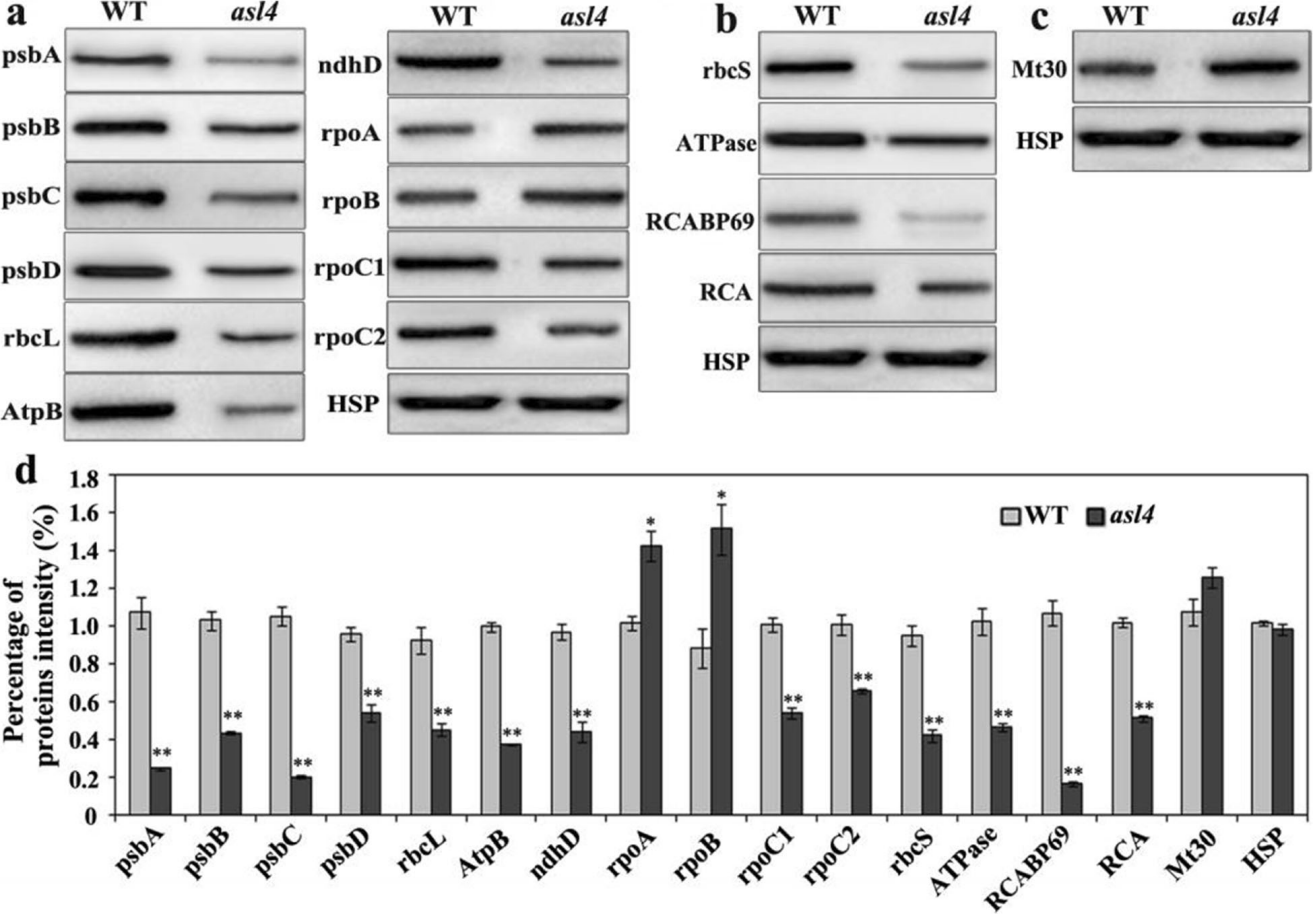
Levels of selected representative proteins. **a** Western blot analysis of plastid-encoded proteins in wild-type and *asl4* mutant seedlings at the L3 stage. **b** Western blot analysis of nuclear-encoded proteins in wild-type and *asl4* mutant seedlings at the L3 stage. **c** Western blot analysis of mitochondrial-encoded proteins in wild-type and *asl4* seedlings at the L3 stage. HSP 82 was used as internal control. **d** Quantification of the band intensity of the detected proteins in *asl4* mutant compare to wild type corresponding to **a**-**c**. Data are means ± SD of three independent repeats. ** and *, indicate significance at *P* = 0.01 and *P* = 0.05, respectively, by Student’s *t* tests

As ASL4 is a chloroplast ribosomal protein we determined whether mutation of the *ASL4* allele affected the synthesis of plastid-encoded proteins by western blot analyses. The contents of most tested plastid-encoded proteins (psbA, psbB, psbC, psbD, rbcL, AtpB, ndhD and rpoC1) were reduced in *asl4* mutant seedlings (Fig. 8a, d). The increased levels of plastid-encoded proteins rpoA and rpoB might be due to increased expression of both genes, or accumulated gene product (Fig. 6a; Fig. 7; Fig. 8a, d). The levels of nuclear-encoded proteins, including rbcS, ATPase, RCABP69 and RCA, were lower (Fig. 8b, d). However, the synthesis of mitochondrial-encoded protein, Mt30, was not affected (Fig. 8c, d). Therefore, we inferred that mutation of *ASL4* had a suppressive role in the synthesis of plastid-encoded proteins.

### The *asl4* Mutant Is Defective in Biogenesis of Chloroplast Ribosomes

**Fig. 9:**
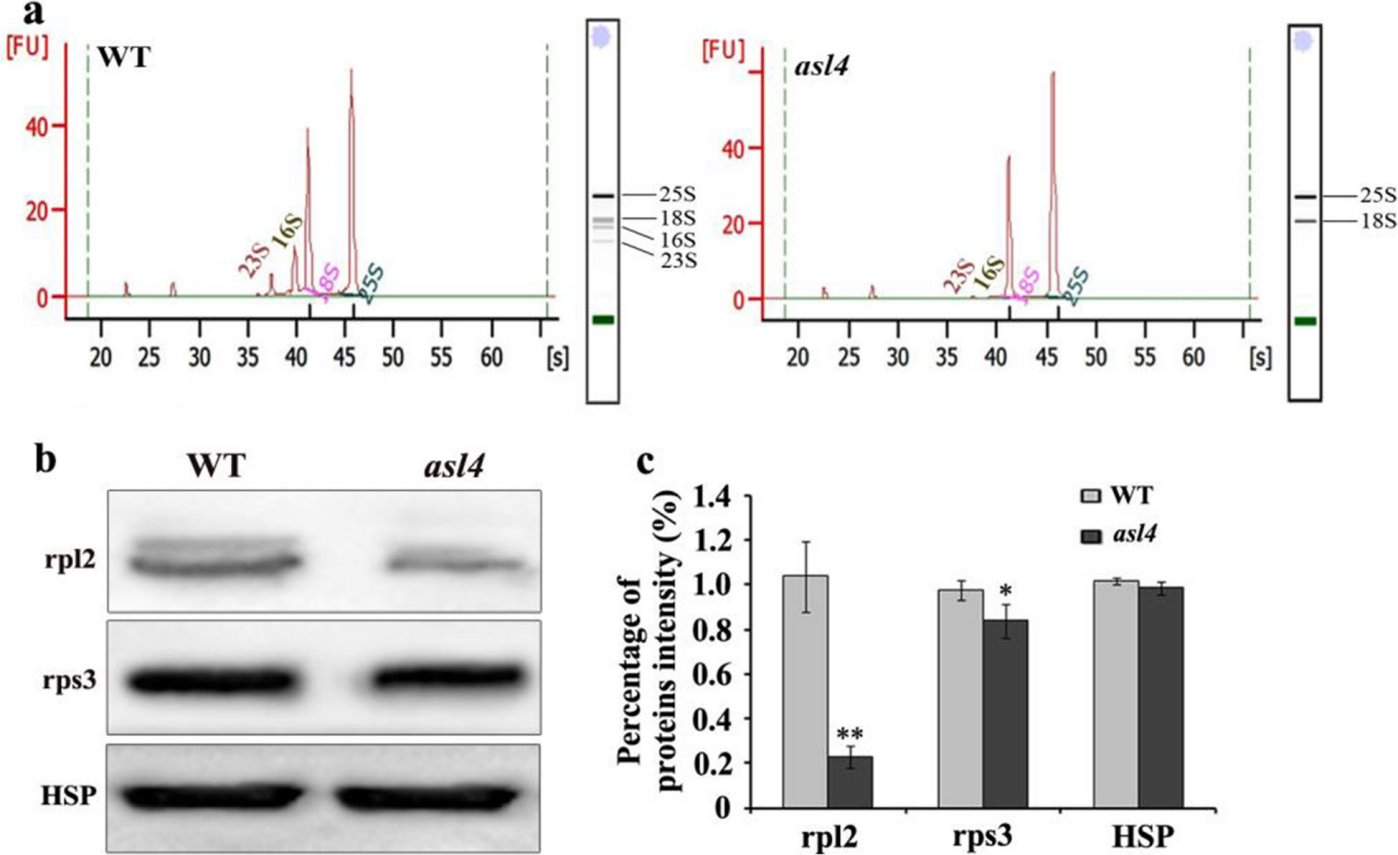
Plastid rRNA and ribosomal protein levels in wild-type and *asl4* mutant seedlings. **a** rRNA analysis of wild-type and *asl4* mutant seedlings at the L3 stage using an Agilent 2100 analyzer. **b** Western blot analysis of plastid ribosomal proteins in wild-type and *asl4* mutant seedlings at the L3 stage. HSP 82 was used as internal control. **c** Quantification of the band intensity of plastid ribosomal proteins corresponding to **b**. Data are means ± SD of three independent repeats. ** and *, indicate significance at *P* = 0.01 and *P* = 0.05, respectively, by Student’s *t* testsl

Chloroplast ribosomes are comprised of 50S large subunits and 30S small subunits, and consist of rRNA (5S, 16S and 23S) and ribosomal proteins. To investigate the changes in chloroplast ribosomes in the *asl4* mutant, we analyzed the components and content of rRNAs using an Agilent 2100 analyzer. The 16S and 23S rRNAs were dramatically decreased in the *asl4* mutant compared to the wild type, whereas the rRNAs in the mitochondrial ribosomes, including 18S and 25S, were not different (Fig. 9a). We also determined that the levels of plastid-encoded ribosomal proteins, including rpl2 (ribosomal protein L2) and rps3 (ribosomal protein S3), were reduced in the *asl4* mutant by western blot analysis (Fig. 9b, c). These results indicated that the biogenesis of chloroplast ribosomes was disrupted in the *asl4* mutant.

## Discussion

Many mutants causing albinism or reduced pigment levels have been reported in rice. The mutants have similar phenotypes including decreased pigment levels, suppressed chloroplast biogenesis and early seedling lethality although caused by different genes. Most of the genes affect components of chloroplast ribosomal proteins (Gong et al. 2013; Lin et al. 2014; Zhao et al. 2016). Here, we identified a new chloroplast ribosomal protein ASL4/PRPS1 in rice, mutation of which caused albinism. Sequence analysis showed that the single copy of OsPRPS1 contained a conserved RNA-binding domain (Fig. S2; S3). The deleted 2855 bases resulted in an incomplete RNA-binding domain in PRPS1 (Fig. S1). Complementation tests verified that an intact RNA-binding domain was responsible for the wild-type phenotype (Fig. 3a, b). Hence PRPS1 is essential for plant growth and development.

*ASL4* transcripts gradually accumulated with elongation of the fourth leaf and reached peak levels in 8-cm L4 leaves at the L3 stage and were also abundant in mature L3 leaves (Fig. 5a, b). Previous study showed that the P4 stage of leaf development corresponded to the three steps of chloroplast biogenesis, including plastid division and DNA replication, establishment of the plastid genetic system, and activation of the photosynthetic apparatus (Kusumi et al. 2010). The first step of chloroplast differentiation was almost complete in 2-cm sections of the fourth leaf in which *ASL4* had higher expression levels than the shoot base (SB) (Fig. 5b). The mRNAs of genes *rpoTp* and *rpoA* involved in the second step of chloroplast differentiation were highly accumulated before 4-cm sections of the fourth leaf. The *rbcL* and *psbA* transcripts involved in the third step were abundant in the later P4 stage, and peaked at the P5 stage (Kusumi et al. 2011). However, *ASL4* was highly expressed in 2 to 8-cm sections of L4 and the L3 leaf (Fig. 5a, b). These observations suggested that *ASL4* might function throughout all three steps of chloroplast differentiation. The reduced mRNA levels of PEP-dependent photosynthesis genes (such as *psaB*, *psbA* and *rbcL*) and increased NEP-dependent housekeeping genes (*rpoA* and *rpoB*) demonstrated that PEP activity was impaired (Fig. 6a, b). This was similar to *Obgc* and chloroplast nucleoid protein-related genes (Bang et al. 2012; Pfalz and Pfannschmidt 2013; Zhong et al. 2013; Zhou et al. 2017). Compromised PEP activity in the *asl4* mutant led to higher expression levels of genes transcribed by NEP and lower levels of genes transcribed by PEP. These genes also participated in the second and third steps of chloroplast biogenesis (Fig. 6a, b). This indicated that impaired PEP activity restrained chloroplast biogenesis in the *asl4* mutant. In addition to increased levels of housekeeping genes, most plastid-encoded genes were down-regulated in the *asl4* mutant (Fig. 7), suggesting that PEP transcription was suppressed. *ASL4* transcripts continuously accumulated with time of illumination during light-induced greening of wild-type seedlings following development in darkness (Fig. 5d), suggesting that synthesis of the chloroplast ribosomal machinery was required for light-induced *ASL4* expression (Merendino et al. 2003).

Proteins are synthesized in three cell compartments, including cytosol, chloroplast and mitochondria. Nuclear-encoded chloroplast ribosomal proteins, which play essential roles in plastidic protein synthesis, need to be post-translationally targeted to the chloroplasts (Schultes et al. 2000; Song et al. 2014). In this study, ASL4 was identified as nuclear-encoded chloroplast ribosomal protein S1 that affects translation by recognizing and modulating most plastid mRNAs in ribosomes. A deficient RNA binding domain made the S1 protein incapable of binding to the ribosome (Hajnsdorf and Boni 2012). The incomplete RNA binding domain in the *asl4* mutant was similarly defective in protein translation. Most plastid-encoded proteins are similarly severely reduced in the *asl4* mutant (Fig. 8a). However, levels of nuclear-encoded proteins were also reduced (Fig. 8b). Chloroplast development depends on the synergism of nuclear and plastid genes. The status of the chloroplast affects transcription of nuclear genes through retrograde signaling (Nott et al. 2006; Liu et al. 2018). The disrupted chloroplast genetic system might therefore restrain the expression and translation of nuclear genes as we found that expression of nuclear-encoded plastid genes was suppressed in the *asl4* mutant (Fig. S5). The plastid ribosome is composed of 50S large and 30S small subunits, and is mainly responsible for translation of plastid proteins. 23S and 5S rRNAs binds to ribosomal large subunit proteins to form 50S large subunits, whereas 16S rRNA binds to ribosomal small subunit proteins to form 30S small subunits (Sharma et al. 2007). Decreased levels of plastid rRNAs and proteins inhibit basal ribosome activity (Aulin et al. 1993; Wang et al. 2016; Zhao et al. 2016). Here, we found that chloroplast 16S and 23S rRNAs were much reduced in the *asl4* mutant, whereas levels of mitochondrial 18S and 25S rRNAs were unchanged (Fig. 9a). The levels of plastid-encoded ribosomal proteins rpl2 and rps3 were also reduced in the mutant (Fig. 9b, c) further inhibiting chloroplast ribosome biogenesis and suppressing synthesis of plastid proteins.

Arabidopsis PRPS1 affects plant growth and photosynthesis (Romani et al. 2012). A T-DNA insertion mutant of *AtPRPS1* showed pale green leaves and reduced plant size but could complete the entire life cycle. However, our mutant of PRPS1 in rice led to different results. Although there were similar reductions in plastid proteins (such as PsbA, PsbB and PsbC) in the rice *asl4* mutant and the Arabidopsis T-DNA insertion line, *psbA*, *rbcL* and *psaB* transcripts were up-regulated in the Arabidopsis mutant whereas the reverse situation was evident in rice (Figs. 7 and 8; Romani et al. 2012). The effects of homologous PRPS1 in Arabidopsis and rice perhaps differed because 8% of *PRPS1* transcripts in the Arabidopsis mutant formed normal protein. The variation in Arabidopsis *PRPS1* appeared not to affect basal ribosome activity, because the 16S and 23S rRNAs were normal in the *AtPRPS1* T-DNA mutant (Romani et al. 2012). However, in this study, we confirmed that OsPRPS1 is essential for plant development. Therefore, we speculate that the *ASL4* mutation reduced the levels of plastid rRNAs and ribosomal proteins, further inhibiting chloroplast ribosome biogenesis, PEP transcription and synthesis of plastid proteins, thereby hindering chloroplast differentiation and photosynthetic pigment synthesis, and ultimately causing albinism.

## Conclusions

Rice *ASL4* encodes 30S ribosomal protein S1, which is targeted to the chloroplast. *ASL4* is essential for chloroplast ribosome biogenesis and early chloroplast development. These data will facilitate efforts to further elucidate the molecular mechanism of chloroplast biogenesis.

## Materials and Methods

### Plant Materials and Growing Conditions

The albino seedling *asl4* mutant was obtained from a MNU-treated population of *Oriza sativa* spp. *japonica* variety Nongyuan 238. The mutant is maintained as a heterozygote. Plants were grown in a paddy field or a growth chamber. The *asl4* mutant plants were studied from the leaf 2 (L2) to leaf 4 (L4) stage. Selected F_2_ populations from a cross between the *asl4* mutant and Nanjing 11 were used to map the *ASL4* locus. For light-induced tests, wild-type seedlings were grown in darkness at 30 °C for 10 days, and were then transferred to light for 24 h (30 °C). Leaf samples were collected every 3 h.

### Confocal, Determination of Photosynthetic Pigments and TEM

A number of Chl-containing cells were investigated in leaves of *asl4* mutant and wild type at the L3 stage by confocal laser scanning microscopy (Carl Zeiss LSM700). Fresh leaves for pigment analysis were collected from L2 and L3 leaves of the *asl4* mutant and wild type as described previously (Zhou et al. 2013). Absorbance was measured with a DU 800 UV/Vis Spectrophotometer (Beckman Coulter). Transverse sections of *asl4* mutant and wild-type leaves for transmission microscopy were prepared from L3 stage leaves of seedlings grown in a paddy following methods previously reported (Zhou et al. 2017). Chloroplast ultrastructure was observed with a Hitachi H-7650 transmission electron microscope.

### Map-Based Cloning and Complementation Test

Ninety two albinic individuals obtained from an *asl4* heterozygous plant (*ASL4*/*asl4*)/Nanjing 11 F_2_ population were used for linkage analysis. A further 1137 albinic F_2_ seedlings were used in fine mapping. New InDel markers were designed with Primer Premier 5.0 based on sequence differences between *indica* and *japonica*. Primers KG-1, KG-2 and KG-3 were used to detect deletions of genomic and cDNA sequences in *asl4* mutant (Suppl. Table S1). Genomic DNAs of three ORFs in the mapping region were amplified and sequenced to detect mutation sites.

For the complementation test, a 6883-bp genomic sequence of the wild-type *ASL4* allele, including a 2513-bp upstream sequence, the *ASL4* coding region and a 407-bp downstream sequence, was amplified from Nongyuan 238 and cloned into binary vector pCAMBIA1305 to generate plasmid p*GASL4*. This vector was then transformed into calli identified as homozygous genotype *asl4asl4* by molecular marker ‘KF’ from selfed progenies of an *ASL4*/*asl4* heterozygote. The empty pCAMBIA1305 vector was also transformed as the control. Marker ‘KF’ was designed to distinguish positive and negative transgenic plants (Suppl. Table S1).

### Bioinformatics Analysis and Subcellular Localization

Candidate genes in the mapping region, sequence information, gene function, and the RNA binding domain of ASL4 were predicted from the RGAP database (http://rice.plantbiology.msu.edu/cgi-bin/gbrowse/rice/). Homologous sequences of the ASL4 protein were identified using NCBI (http://www.ncbi.nlm.nih.gov/) and sequences were aligned using BioEdit software. A neighbor-joining tree based on 1000 bootstrap replicates was performed with MEGA v4.1 software. The expression profile of *ASL4* gene was predicted with the RiceXPro database (http://ricexpro.dna.affrc.go.jp/).

For subcellular localization, a 1206-bp coding sequence without the TAG stop codon of the *ASL4* allele was cloned into the N-terminus of GFP in the pA7 vector, which was then transiently transformed into rice protoplasts. The empty vector was similarly transformed as the control. Fluorescence was observed using the confocal laser scanning microscope (Carl Zeiss LSM700).

### Gene Expression Analysis

Total RNA was extracted using RNA Prep Pure Plant kit (TIANGEN) and reverse-transcribed with a FastKing RT Kit (TIANGEN) according to the manufacturer’s instructions. Quantitative RT-PCR was performed using a SYBR_ Premix Ex TaqTM kit (TaKaRa) on an ABI Q3 Real-Time PCR System. Relative gene expression was analyzed using 2^-ΔΔCT^ method (Livak and Schmittgen 2001). Primers for quantitative Real-Time PCR were designed by Primer Premier 5.0 or GenScript. The *ubiquitin* gene (ubq) was used as a reference (Suppl. Table S1).

### Transcriptome Analysis

Total RNA was isolated from L3 seedlings of the *asl4* mutant and wild type. RNA purity was tested with a Nanodrop and RNA integrity and contents of rRNAs were detected by Agilent 2100 analyzer. A library was constructed and sequenced with an lllumina HiSeq 2000 (Novogene). Data were analyzed by the RPKM method (Mortazavi et al. 2008). Plastid-encoded genes were isolated by referring to the chloroplast genome annotation (http://megasun.bch.umontreal.ca/ogmp/projects/other/cp_list.html). Genes with significant differences in expression were determined (*P* value < 0.05, log_2_ (FoldChange) > 1 or < − 1).

### Western Blot Analysis

Total proteins were extracted from wild-type and *asl4* mutant seedlings at the L3 stage. Tissue samples were ground in liquid nitrogen and isolated with equal volumes of NB1 solution (50 mM Tris-Mes, 0.5 M sucrose, 1 mM MgCl_2_, 10 mM EDTA, 5 mM DTT and protease inhibitor cocktail CompleteMini tablets, pH 8.0) on ice at 20 rpm for 30 min. The supernatant was collected by centrifugation at 12,000 rpm for 10 min and denatured by adding 5× loading buffer at 95 °C for 5 min. The proteins were separated in SDS-PAGE gels, transferred to polyvinylidene difluoride membranes, and identified with antibodies using an ECL Plus Western Blotting Detection Kit (Thermo). Proteins were quantified by Quantity One software. The relevant antibodies were obtained from BPI (http://www.proteomics.org.cn/).

## Supplementary Information

**Additional file 1: Supplemental figure S1.** Sequence alignment of the ASL4 and asl4 proteins. *Black underline* indicates the RNA binding domain. **Supplemental figure S2.** Sequence alignment of ASL4-related proteins. Sequences are for OsPRPS1 (OsASL4, *Oryza sativa*, LOC_Os03g20100), BdPRPS1 (*Brachypodium distachyon*, XP_003558047.1), TuPRPS1 (*Triticum urartu*, EMS48000.1), SbPRPS1 (*Sorghum bicolor*, XP_002465357.1), SiPRPS1 (*Setaria italic*, XP_004984580.1), ObPRPS1(*Oryza brachyantha*, XP_006649986.1), ZmPRPS1 (*Zea mays*, AQL07040.1), BnPRPS1 (*Brassica napus*, XP_013644063.1), AtPRPS1 (*Arabidopsis thaliana*, NP_850903.1), GmPRPS1 (*Glycine max*, NP_001348025.1), PpPRPS1 (*Physcomitrella patens*, XP_024386702.1), PsPRPS1 (*Picea sitchensis*, ABK25672.1). Red underline indicates the RNA binding domain. **Supplemental figure S3.** Phylogenetic analysis of ASL4 and its related proteins. OsPRPS1 is indicated a *black asterisk*. Sequences are for OsPRPS1 (OsASL4, *Oryza sativa*, LOC_Os03g20100), PpPRPS1 (*Physcomitrella patens*, XP_024386702.1), PsPRPS1 (*Picea sitchensis*, ABK25672.1). NnPRPS1(*Nelumbo nucifera*, XP_010270863.1), PdPRPS1(*Phoenix dactylifera*, XP_008781183.1), VvPRPS1(*Vitis vinifera*, XP_002280604.1), MnPRPS1(*Morus notabilis*, XP_010102913.1), TcPRPS1(*Theobroma cacao*, XP_017975185.1), LsPRPS1(*Lactuca sativa*, XP_023760774.1), AtPRPS1 (*Arabidopsis thaliana*, NP_850903.1), SoPRPS1(*Spinacia oleracea*, XP_021854510.1), GmPRPS1 (*Glycine max*, NP_001348025.1), ObPRPS1(*Oryza brachyantha*, XP_006649986.1), BdPRPS1 (*Brachypodium distachyon*, XP_003558047.1), TuPRPS1 (*Triticum urartu*, EMS48000.1), SbPRPS1 (*Sorghum bicolor*, XP_002465357.1), ZmPRPS1 (*Zea mays*, AQL07040.1), PmPRPS1(*Panicum miliaceum*, RLN42086.1), SiPRPS1 (*Setaria italic*, XP_004984580.1). **Supplemental figure S4.** Expression profile of *ASL4* at different growth stages. Colors represent different tissues. Data were analyzed in RiceXPro, the rice expression profile database. **Supplemental figure S5.** Expression levels of genes associated with Chlorophyll biosynthesis in wild-type and *asl4* mutant seedlings at the L3 stage. Data are means ± SD of three independent repeats. **, significance at *P* = 0.01 when analyzed by Student’s *t* tests.

**Additional file 2: Table S1.** Primer sequences used in this study

## Data Availability

All data supporting the conclusions of this article are provided within the article (and its additional files).

## Abbreviations

*asl4*: *albino seedling lethality 4*
Car: Carotenoids
Chl: Chlorophyll
GFP: Green fluorescent protein
GUN1: Genomes uncoupled 1
InDel: Insertion-deletion polymorphic
MNU: *N*-methyl-*N*-nitrosourea
NEP: Nuclear-encoded phage-type RNA polymerase
ORF: Open reading frame
PEP: Plastid-encoded plastid RNA polymerase
PRPL: Plastid ribosomal protein large subunit
PRPS: Plastid ribosomal protein small subunit
SB: Shoot base
TEM: Transmission electron microscopy
WLP1: White leaf and panicles 1
