# Supplementary material for: Albino seedling lethality 4; Chloroplast 30S Ribosomal Protein S1 is Required for Chloroplast Ribosome Biogenesis and Early Chloroplast Development in Rice
Source: Rice (N Y). 2021 May 27;14:47. doi: 10.1186/s12284-021-00491-y (PMC8160077; doi:10.1186/s12284-021-00491-y)
Supplement: Supplementary file 1 — Additional file 1: Supplemental figure S1. Sequence alignment of the ASL4 and asl4 proteins. Black underline indicates the RNA binding domain. Supplemental figure S2. Sequence alignment of ASL4-related proteins. Sequences are for OsPRPS1 (OsASL4, Oryza sativa, LOC_Os03g20100), BdPRPS1 (Brachypodium distachyon, XP_003558047.1), TuPRPS1 (Triticum urartu, EMS48000.1), SbPRPS1 (Sorghum bicolor, XP_002465357.1), SiPRPS1 (Setaria italic, XP_004984580.1), ObPRPS1(Oryza brachyantha, XP_006649986.1), ZmPRPS1 (Zea mays, AQL07040.1), BnPRPS1 (Brassica napus, XP_013644063.1), AtPRPS1 (Arabidopsis thaliana, NP_850903.1), GmPRPS1 (Glycine max, NP_001348025.1), PpPRPS1 (Physcomitrella patens, XP_024386702.1), PsPRPS1 (Picea sitchensis, ABK25672.1). Red underline indicates the RNA binding domain. Supplemental figure S3. Phylogenetic analysis of ASL4 and its related proteins. OsPRPS1 is indicated a black asterisk. Sequences are for OsPRPS1 (OsASL4, Oryza sativa, LOC_Os03g20100), PpPRPS1 (Physcomitrella patens, XP_024386702.1), PsPRPS1 (Picea sitchensis, ABK25672.1). NnPRPS1(Nelumbo nucifera, XP_010270863.1), PdPRPS1(Phoenix dactylifera, XP_008781183.1), VvPRPS1(Vitis vinifera, XP_002280604.1), MnPRPS1(Morus notabilis, XP_010102913.1), TcPRPS1(Theobroma cacao, XP_017975185.1), LsPRPS1(Lactuca sativa, XP_023760774.1), AtPRPS1 (Arabidopsis thaliana, NP_850903.1), SoPRPS1(Spinacia oleracea, XP_021854510.1), GmPRPS1 (Glycine max, NP_001348025.1), ObPRPS1(Oryza brachyantha, XP_006649986.1), BdPRPS1 (Brachypodium distachyon, XP_003558047.1), TuPRPS1 (Triticum urartu, EMS48000.1), SbPRPS1 (Sorghum bicolor, XP_002465357.1), ZmPRPS1 (Zea mays, AQL07040.1), PmPRPS1(Panicum miliaceum, RLN42086.1), SiPRPS1 (Setaria italic, XP_004984580.1). Supplemental figure S4. Expression profile of ASL4 at different growth stages. Colors represent different tissues. Data were analyzed in RiceXPro, the rice expression profile database. Supplemental figure S5. Expression levels of genes associated with Chloroph [file 12284_2021_491_MOESM1_ESM.docx]

.
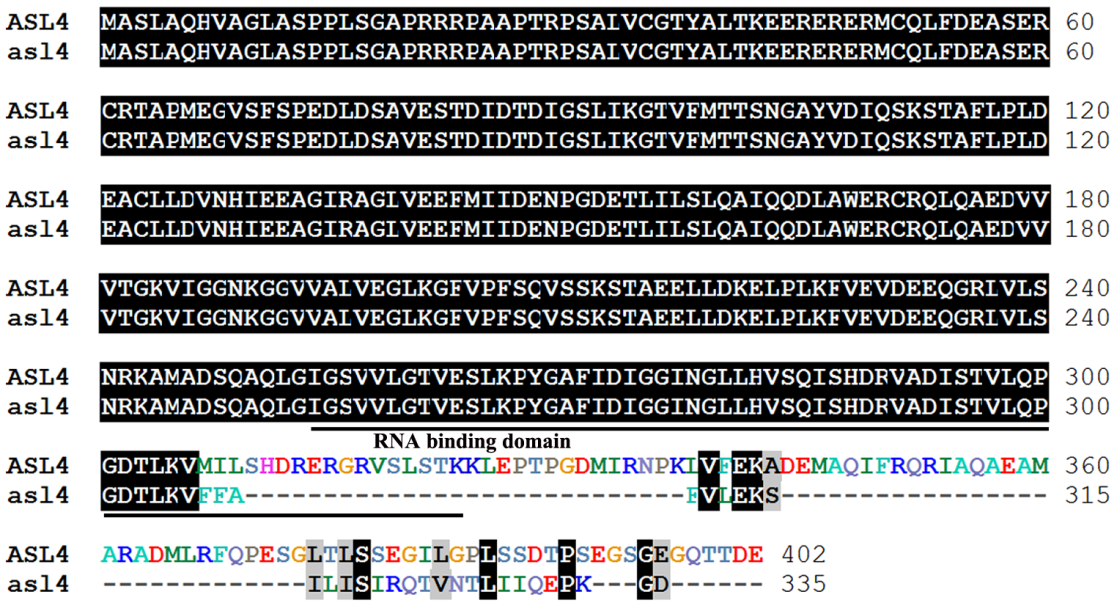


Supplemental figure S1 Sequence alignment of the ASL4 and asl4 proteins. *Black underline* indicates the RNA binding domain.


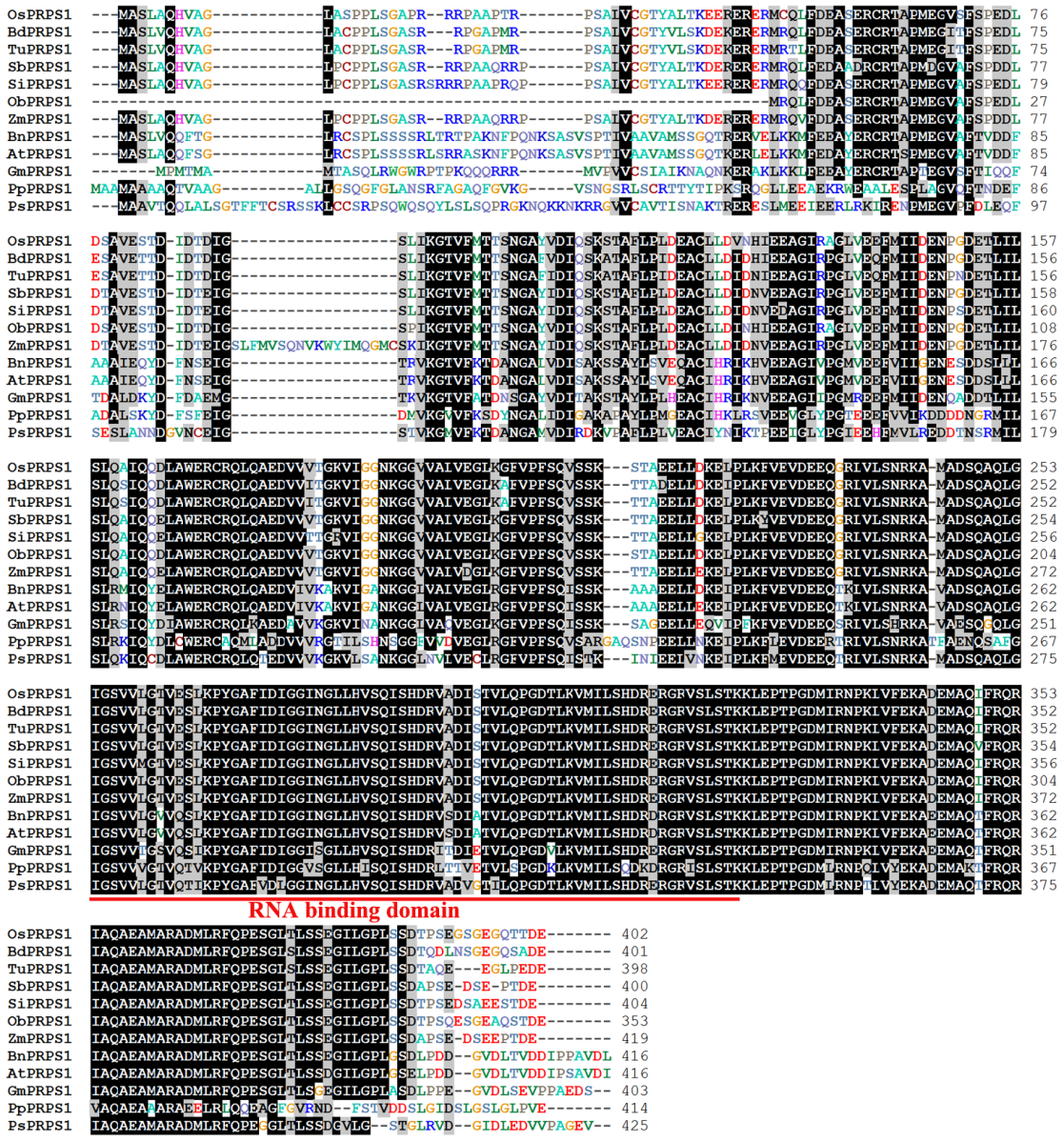


Supplemental figure S2 Sequence alignment of ASL4-related proteins. Sequences are for OsPRPS1 (OsASL4, *Oryza sativa*, LOC_Os03g20100), BdPRPS1 (*Brachypodium distachyon*, XP_003558047.1), TuPRPS1 (*Triticum urartu*, EMS48000.1), SbPRPS1 (*Sorghum bicolor*, XP_002465357.1), SiPRPS1 (*Setaria italic*, XP_004984580.1), ObPRPS1(*Oryza brachyantha*, XP_006649986.1), ZmPRPS1 (*Zea mays*, AQL07040.1), BnPRPS1 (*Brassica napus*, XP_013644063.1), AtPRPS1 (*Arabidopsis thaliana*, NP_850903.1), GmPRPS1 (*Glycine max*, NP_001348025.1), PpPRPS1 (*Physcomitrella patens*, XP_024386702.1), PsPRPS1 (*Picea sitchensis*, ABK25672.1). Red underline indicates the RNA binding domain.


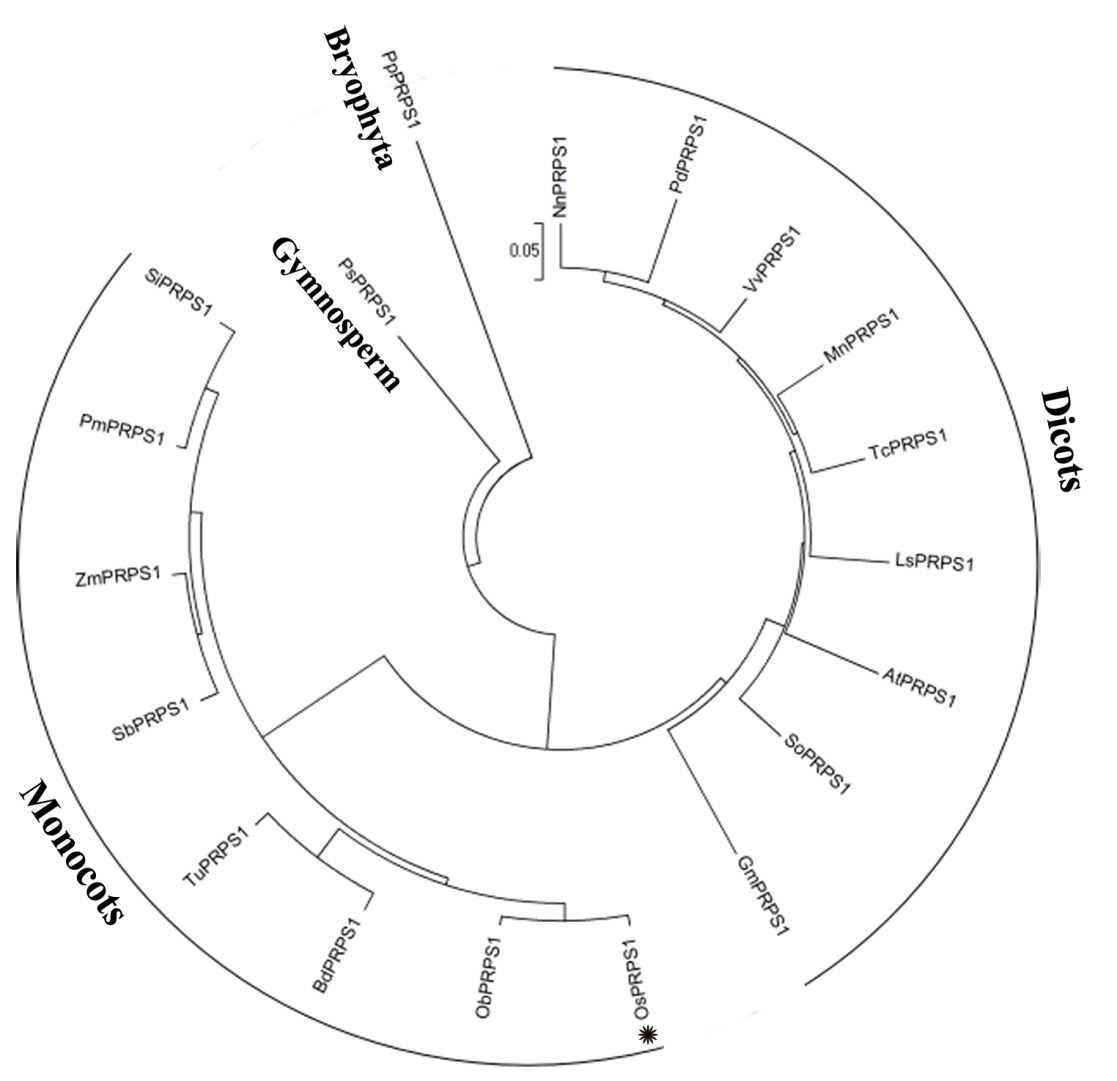


Supplemental figure S3 Phylogenetic analysis of ASL4 and its related proteins. OsPRPS1 is indicated a *black asterisk*. Sequences are for OsPRPS1 (OsASL4, *Oryza sativa*, LOC_Os03g20100), PpPRPS1 (*Physcomitrella patens*, XP_024386702.1), PsPRPS1 (*Picea sitchensis*, ABK25672.1). NnPRPS1(*Nelumbo nucifera*, XP_010270863.1), PdPRPS1(*Phoenix dactylifera*, XP_008781183.1), VvPRPS1(*Vitis vinifera*, XP_002280604.1), MnPRPS1(*Morus notabilis*, XP_010102913.1), TcPRPS1(*Theobroma cacao*, XP_017975185.1), LsPRPS1(*Lactuca sativa*, XP_023760774.1), AtPRPS1 (*Arabidopsis thaliana*, NP_850903.1), SoPRPS1(*Spinacia oleracea*, XP_021854510.1), GmPRPS1 (*Glycine max*, NP_001348025.1), ObPRPS1(*Oryza brachyantha*, XP_006649986.1), BdPRPS1 (*Brachypodium distachyon*, XP_003558047.1), TuPRPS1 (*Triticum urartu*, EMS48000.1), SbPRPS1 (*Sorghum bicolor*, XP_002465357.1), ZmPRPS1 (*Zea mays*, AQL07040.1), PmPRPS1(*Panicum miliaceum*, RLN42086.1), SiPRPS1 (*Setaria italic*, XP_004984580.1)


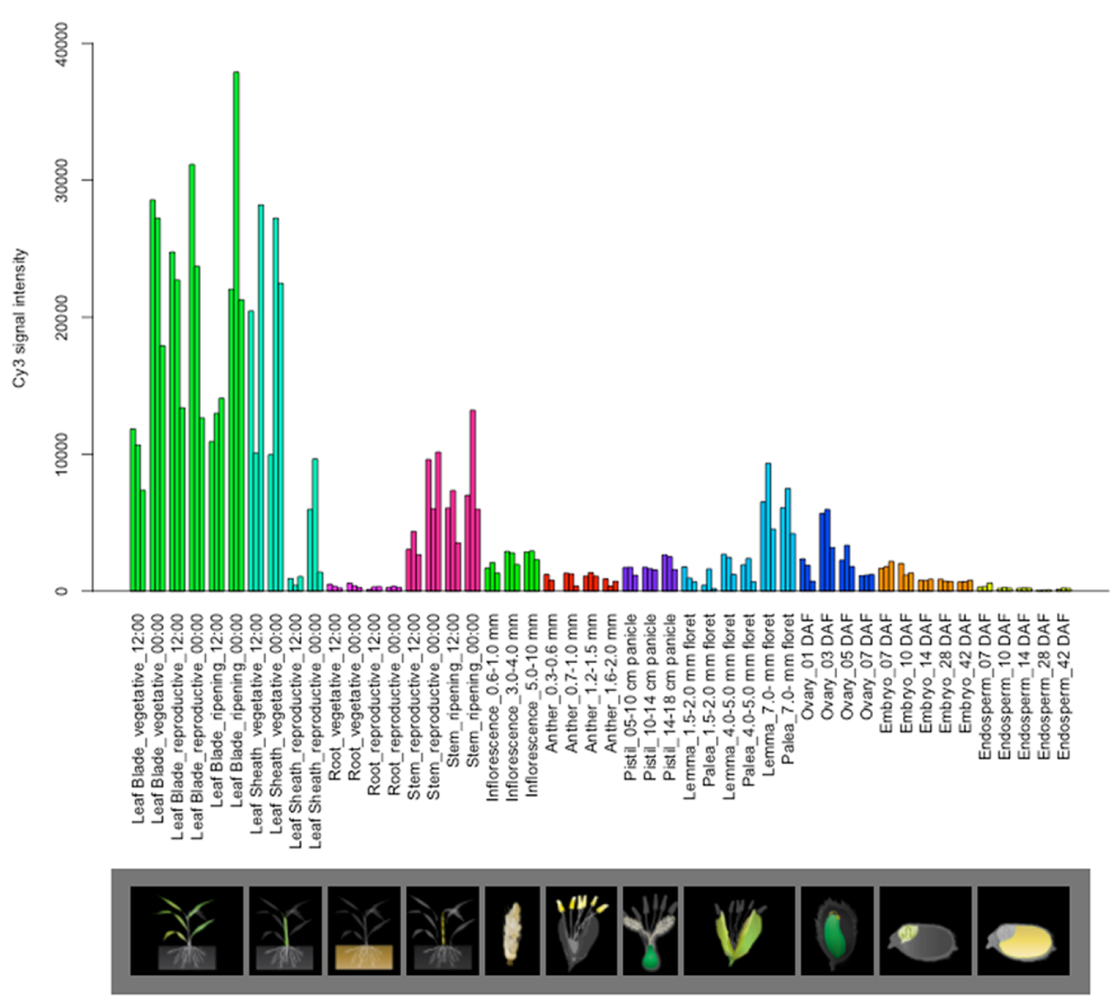


Supplemental figure S4 Expression profile of *ASL4* at different growth stages. Colors represent different tissues. Data were analyzed in RiceXPro, the rice expression profile database.


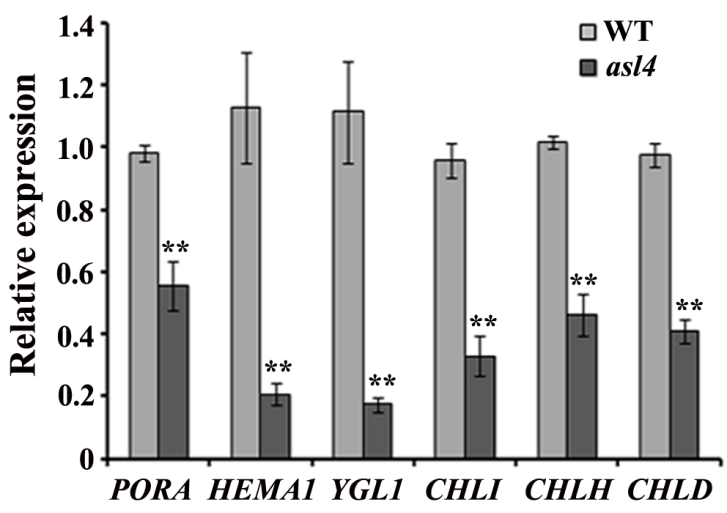


Supplemental figure S5 Expression levels of genes associated with Chlorophyll biosynthesis in wild-type and *asl4* mutant seedlings at the L3 stage. Data are means ± SD of three independent repeats. **, significance at *P* = 0.01 when analyzed by Student’s *t* tests.
