# Supplementary material for: Albino seedling lethality 4; Chloroplast 30S Ribosomal Protein S1 is Required for Chloroplast Ribosome Biogenesis and Early Chloroplast Development in Rice
Source: Rice (N Y). 2021 May 27;14:47. doi: 10.1186/s12284-021-00491-y (PMC8160077; doi:10.1186/s12284-021-00491-y)
Supplement: Supplementary file 2 — Additional file 2: Table S1. Primer sequences used in this study [file 12284_2021_491_MOESM2_ESM.pdf]

Table S1. Primer sequences used in this study

Mapping and detecting primers		
Name	Forward sequence (5'-3')	Reverse sequence (5'-3')
C3-16	CGAGGACAGCAGGAATAAGG	AGTTTACGAGTTGCCAAGCA
K5	CGGACTCCTCTGATACGATA	GGCATTTTGCACCATACA
K20	GGTTTCAGATTTGCCACG	TTCATTCCGCATTTGGTC
K22	ACATCTGAATCTCACAACCGAA	AAAGATCCAAGGAGTATGACGA
K27	TTGAGGGATACGATTTAGATTC	ATTCGACCTGCCCGTTAGT
K29	CCAACCGTTTCTACGTTGACT	TGTAGCCCGTATGCTCTTCTC
K31	TTTACTCGCCGCTTTGGAC	CAGCAGGGAGGATTGGGAT
K40	CGGACGACGAGGCGAACC	AGGAGCAGCGTGCGGCTG
KG-1	CACTAGCTGCCCACTATTTTAC	TGATACAAGCACAGATCGAAGA
KG-2	TGTGGAGGTCGATGAGGAA	GAGGGAGTATAAGATTGGTGAGAA
KG-3	TTGTTGTCACGGGTAAAGTA	CCTGAATATCTGAGCCATCT
KF	TGCAACCAGGAGATACGCTCA	CTAAGATGCCCTCTGAACTGA
ACTIN	ACGGAGCGTGGTTACTCATTC	ATCGCAACTCAAAAACAACCAG
Quantitative RT-PCR Primers		
Name	Forward sequence (5'-3')	Reverse sequence (5'-3')
Ubq	GCTCCGTGGCGGTATCAT	CGGCAGTTGACAGCCCTAG
ASL4-RT	GCTGGTGTGCGGGACGTA	CTCGGAGGCCTCGTCGAAC
FtsZ	GTTGGTGTTTCTTCCAGCAA	CCTCAATAGACGACCCGATT
rpoTp2	AAGTCTGGCTTACGCTGGTT	AGGATCCTCAGCATTCATCC
rpoA	AAATCGTTGATACGGCACAA	ATTCACATTTCGAACAGGCA
rpoB	GCATTGTTGGAACTGGATTG	GCCGATGGGTAACTAAAGGA
rbcL	GTTGAAAGGGATAAGTTGA	AATGGTTGTGAGTTTACG
psbA	AAGTTTCTCTGATGGTATG	ATAGCACTGAATAGGGAA
psaB	TTGGTATTGCTACCGCACAT	CCGGACGTCCATAGAAAGAT
psbB	TCATATTGCTGCGGGTACAT	AGTTGCTGACCCATACCACA
psbC	TACAACCTTGGCAAGAACGA	TACGCCACCCACAGAATTTA
rbcS	TCATCAGCTTCATCGCCTAC	ACTGGGAACACACGAAACAA
cab1R	AGACGTTCGCCAAGAACC	GAGGAGCTCCGGGAAGAC
cab2R	GTTCTCCATGTTCGGCTTCT	GACGAAGTTGGTGGCGTAG
PORA	ATCACCAAGGGCTACGTCTC	GAGTTGTTGTTCCAGCTCCA
HEMA1	CACCAGTCTGAATCATAT	CTACCACTTCTCTAATCC
YGL1	TGGACAGTTGAAGATGTT	GAATAGGACGGTAAGGTT
CHLI	AGTAACCTTGGTGCTGTG	AATCCATCAACATTCAACTCTG
CHLH	CTATACATTCGCCACACT	TATCACACAACTCCCAAG
CHLD	GGAAAGAGAGGGCATTAG	CAATACGATCAAGTAAGTGTT

Vector construction primers		
Name	Primer pairs (5'-3')	Restriction enzyme
ASL4genomics	CCATGATTACGAATTCCTGTAGCCCGTATGCTCTTCTC	*EcoR I*
	CCAAGCTTGCATGCCTGCAGTGGCTTCCGTGAACTACTTGC	*Pst I*
ASL4-GFP	GGACTAGTATGGCGTCCCTGGCGCAGCA	*Spe I*
	GCTCTAGATTCATCTGTGGTTTGCCCTTCTC	*XbaI*
